# Plant genetic resources in India: management and utilization

**DOI:** 10.18699/VJ20.622

**Published:** 2020-05

**Authors:** K. Singh, K. Gupta, V. Tyagi, S. Rajkumar

**Affiliations:** ICAR-National Bureau of Plant Genetic Resources, Pusa Campus, New Delhi, India; ICAR-National Bureau of Plant Genetic Resources, Pusa Campus, New Delhi, India; ICAR-National Bureau of Plant Genetic Resources, Pusa Campus, New Delhi, India; ICAR-National Bureau of Plant Genetic Resources, Pusa Campus, New Delhi, India

**Keywords:** plant genetic resources, gene banks, wild relatives, biotic and abiotic stresses, marker-assisted selection, генетические ресурсы растений, генетические банки, дикорастущие родственники культурных растений, биотический и абиотический стресс, отбор с помощью маркеров

## Abstract

Plant genetic resources (PGR) are the foundation of agriculture as well as food and nutritional security. The ICAR-NBPGR is the nodal institution at national level for management of PGR in India under the umbrella of Indian Council of Agricultural Research (ICAR), New Delhi. India being one of the gene-rich countries faces a unique challenge of protecting its natural heritage while evolving mutually beneficial strategies for germplasm exchange with other countries. The Bureaus activities include PGR exploration, collection, exchange, characterization, evaluation, conservation and documentation. It also has the responsibility to carry out quarantine of all imported PGR including transgenics meant for research purposes. The multifarious activities are carried out from ICAR-NBPGR headquarters and its 10 regional stations located in different agro-climatic zones of India. It has linkages with international organizations of the Consultative Group on International Agricultural Research (CGIAR) and national crop-based institutes to accomplish its mandated activities. NBPGR collects and acquires germplasm from various sources, conserves it in the Genebank, characterizes and evaluates it for different traits and provides ready material for breeders to develop varieties for farmers. ICAR-NBPGR encompasses the National Genebank Network and at present, the National Genebank conserves more than 0.40 million accessions. NBPGR works in service-mode for effective utilization of PGR in crop improvement programmes which depends mainly on its systematic characterization and evaluation, and identification of potentially useful germplasm. NBPGR is responsible for identifying trait-specific pre-adapted climate resilient genotypes, promising material with disease resistance and quality traits which the breeders use for various crop improvement programmes. The system has contributed immensely towards safeguarding the indigenous and introducing useful exotic PGR for enhancing the agricultural production. Presently, our focus is on characterization of ex situ conserved germplasm and detailed evaluation of prioritized crops for enhanced utilization; assessment of impact of on-farm conservation practices on genetic diversity; genome-wide association mapping for identification of novel genes and alleles for enhanced utilization of PGR; identification and deployment of germplasm/landraces using climate analog data; validation of trait-specific introduced germplasm for enhanced utilization. Key words: plant genetic resources; gene banks; wild relatives; biotic and abiotic stresses; marker-assisted selection.

## Abstract (ru)

Генетические ресурсы растений – основа сельского хозяйства и главный фактор, определяющий качество потребляемой пищи. В Индии на национальном уровне этой проблемой занимается Национальное бюро генетических ресурсов растений (NBPGR), действующее под эгидой Индийского совета по сельскохозяйственным исследованиям (ICAR), со штаб-квартирой в Нью-Дели. Обладая богатыми растительными ресурсами, Индия должна учитывать интересы безопасности своего природного наследия при выработке даже самых выгодных стратегий обмена генетическим материалом со своими международными партнерами. В задачи Бюро входят исследование, сбор, обмен, описание, оценка, сохранение и учет генетических ресурсов растений, а также обеспечение карантинных мер для всего ввозимого из-за рубежа материала, включая трансгенные растения, предназначенные для исследовательских целей. Бюро и десять его региональных отделений, расположенных в разных агроклиматических зонах страны, осуществляют деятельность в нескольких направлениях. Поддерживают связи с международными организациями, входящими в состав Консультативной группы по международным сельскохозяйственным исследованиям(CGIAR), и национальными институтами, занимающимися проблемами сельскохозяйственных культур. Образцы генофонда из самых разных источников пополняют генбанк, где проводится их описание и оценка по заданным признакам. На основе этого материала выводятся сорта сельскохозяйственных культур. Су- ществующий при Бюро Национальный генетический банк (National Genebank Network) насчитывает более 400 тысяч образцов. Бюро работает в сервисном режиме, обеспечивая эффективное использование гене- тических ресурсов растений в программах улучшения сельскохозяйственных культур, что стало возмож- ным во многом благодаря последовательному подходу к описанию и оценке этих ресурсов, а также отбору потенциально полезного генетического материала. Другими задачами являются определение генотипов с теми или иными признаками, специфичными к изменению климата, а также отбор перспективного мате- риала, обладающего устойчивостью к заболеваниям и признаками качества, на которые ориентируются селекционеры при работе над улучшением сельскохозяйственных культур. Действующая таким образом система сыграла важнейшую роль в выработке столь необходимого стране баланса в отношении генетиче- ских ресурсов растений: интродукция ценного экзотического генофонда в целях интенсификации произ- водства сельскохозяйственной продукции ведется без ущерба для местных ресурсов. В настоящее время основными направлениями работы являются: описание генетического материала, сохраненного путем консервации ex situ, и всесторонняя оценка приоритетных сельскохозяйственных культур для более эф- фективного их использования; оценка влияния различных методов мелиорации земель на генетическое разнообразие; полногеномное ассоциативное картирование с целью выявления ранее неизвестных генов и аллелей для более эффективного использования генетических ресурсов растений; отбор генетического материала и/или местных разновидностей и определение оптимальных районов выращивания на основе аналоговых данных наблюдений за климатом; проверка соответствия интродуцированного генетического материала заданным критериям.

## Introduction

Plant genetic resources (PGR) are one of essential components of agro-biodiversity and defined as the genetic material of plants having value as a resource for present and future generations. PGR hold the key to the very foundation of agriculture as well as food and nutritional security for the world. Agricultural biodiversity, agri- or agro-biodiversity (a subset of biodiversity) is defined as ‘all crops and livestocks, their wild relatives, and all interacting species of pollinators, symbionts, pests, parasites, predators and competitors’ (Qualset et al., 1995). Indian subcontinent has a rich and varied heritage of biodiversity, encompassing a wide spectrum of habitats from tropical rainforests to alpine vegetation and from temperate forests to coastal wetlands. It is one of the eight centres of origin (Vavilov, 1951) and is one of the 12 mega gene centres of the world. It possesses 11.9 % of world flora, and about 33 % of the country’s recorded flora are endemic to the region and are concentrated mainly in the North-East, Western Ghats, North West Himalayas and the Andaman and Nicobar islands. Of the 49,219 higher plant species, 5,725 are endemic and belong to 141 genera under 47 families (Nayar, 1980). Of these 3,500 are found in the Himalayas and adjoining regions and 1,600 in the Western Ghats alone (Arora, 1991). The concept of biodiversity hotspots was originated by Dr. Norman Myers in two articles in “The Environmentalist” (1988), revised after thorough analysis by Myers and others in “Hotspots: Earth’s biologically Richest and Most Endangered Terrestrial Ecoregions”. The hotspots idea was also promoted by Russell Mittermeier in the popular book “Hotspots revisited”. Around the world, 34 biodiversity hotspots exists as of today. These sites support nearly 60 % of the world’s plant, bird, mammal, reptile, and amphibian species, with a very high share of endemic species (Myers et al., 2000).

From centuries, tribal or traditional farming communities have continuously adapted and shapened the dimensions of rich genetic material available with them. These resources or traditional varieties or landrace populations often bear specific traits – early or late maturing, adaptability to a particular soil type, uses and usually have local names which has enabled them to survive so long under various biotic and abiotic stresses in the centers of diversity along with wild progenitors of crop plants, wild and weedy relatives. Besides these resources, potential domesticates (also called wild economic species) are involved in the PGR spectrum; they are those wild species, which are not yet domesticated but are extensively used. Some of them grow widely, though genetically and culturally in a near wild state. With richness of plant genetic resources, the tribal regions have therefore been identified as “Hot Spots” of agri-biodiversty. However, they are different from the biodiversity hot-spots (as per definition of Myers et al., 2000) which include all entire endemic biodiversity as a priority for defining hot-spots of a region.

During the process of crop evolution crops originated in its centre of origin have changed from the wild progenitors in morphological, physiological and agronomic traits to newer types through selection for desired traits. In this whole process, agriculture (broadly the process of rearing plants and animals, wild or tamed), cultivation (physical activities which are relevant to and associated with agriculture) and domestication (process of genetic shift in domesticated population to adapt them to better/changed or artificial environment, created by cultivation conditions) have shapened them for appropriate PGR. PGRs are exchanged and searched continuously for specific traits to improve crops in terms of yield and nutritional value, and their interdependence plays a very important role in international collection and exchange of germplasm. Every nation is concerned with acquisition of diverse and superior germplasm for conservation and utilization.

Over the years, India has developed sound and scientific management regimes for ex situ conservation and access to its genetic resources (Dhillon, Saxena, 2003). Groups of institutions, scientific societies, non-governmental organizations are addressing the task with ICAR-NBPGR, New Delhi, as the nodal agency for its coordination. It aims at efficient management of plant genetic resources by providing convenience of access to the various crop improvement programmes. It also encompasses the National Genebank Network. At present, the genebank holds more than 4.4 lakhs accessions. The Cryobank Facility in the National Genebank has accessions of varied germplasm of orthodox, intermediate and recalcitrant seed species and also of pollen samples. The in vitro genebank conserves various priority crops which are maintained under short- to medium-term storage periods. These include tuberous and bulbous crops, tropical fruits species, spices and industrial crops, medicinal and aromatic plants species.

Centre of origin and diversity of crop plants are concepts relating to the patterns of distribution and build-up in regions and habitats, consequent to use and domestication by man in areas representing independent agricultural systems and separated by major geographical barriers. Various terms have been used to designate these ‘geobotanical’ diversity patterns in crop plants viz. primary and secondary centres of diversity, gene centres, cradles/subcradles of agriculture, megacentres, regions (Vavilov, 1926; Zhukovsky, 1968), non-centres, microcentres (Harlan, 1975), cradles of angiosperm diversity (Takhtajan, 1969). However, within centres of diversity, geobotanical patterns of variation, add a practical connotation to the use of these concepts in PGR. The significant point, as stated above is that centres of diversity are the consequence of continuing processes of evolution and domestication, and hence subject to change. The concept of ‘ecological passports’ in different sites where different crops show parallelism in characters by Vavilov, form the basis for understanding the diversity within the crop species, and in relation to the crop genepool (Harlan, de Wet, 1971).

**PGR conservation**

The National Bureau of Plant Genetic Resources (ICARNBPGR) was established by the Indian Council of Agricultural Research (ICAR) in 1976 with its headquarters at New Delhi. The chronology of events leading to the present day ICARNBPGR dates back to 1905 when Botany Division was established under the then Imperial Agricultural Research Institute. ICAR-NBPGR has been given the mandate to act as a nodal institute at the national level for acquisition and management of indigenous and exotic PGR for agriculture, and to carry out related research and human resources development for sustainable growth of agriculture. The Bureau is also vested with the authority to issue Import Permit and Phytosanitary Certificate and conduct quarantine checks in seed material and vegetative propagules (including transgenic material) introduced from abroad or exported for research purposes. Besides having a 40 ha experimental farm at Issapur village (about 45 km west of Pusa Campus), the Bureau has a strong national network comprising Regional Stations/Base Centers and ICAR Institutes/SAUs that provide access to representative agro-ecological situations in the country.

The major components of the National Genebank include the seed genebank, field genebank, cryo-bank and the in vitro genebank. The focus of seed genebank of NGB is long term conservation of orthodox seeds. The basic parameters that are assessed for conservation are:

- Uniqueness of the accession. Redundancy is a major issue in all genebanks and best efforts are made to avoid duplication of accessions.
- Seed quality. The global genebank standards recommends a minimum of 2000 seeds in self-pollinated crops and 4000 seeds in cross pollinated crops. In wild germplasm accessions, the minimum number has been relaxed to 500 seeds.
- Seed viability. A minimum viability of 85 % is essential for seed samples. However in those cases where the Indian Minimum Seed Certification Standards have approved a lower level of standard germination, the viability requirements are accordingly modified in gene bank also.
- Seed health. Pest free conservation is a priority at NGB. There is a strong collaboration between the Division of Germplasm Conservation and Division of Plant Quarantine, wherein all accessions are tested for any form of pest infestation prior to their processing.
- Availability of passport information. The utilization of the conserved accessions can be facilitated only if all relevant passport information is available in the database. Hence, only those accessions having basic information on parameters like biological status, collection details if acquired through exploration, pedigree details if it’s a breeding line/ genetic stock or cultivar and any other unique trait if applicable, is accepted for long term conservation.

The qualified accessions are then subjected to drying, which is the most crucial step in genebank processing. A walk-indrying chamber functioning at 15 % RH and 15 °C is used for the drying purpose. For species with hard seed coats and which require longer drying duration are shifted to batch dryers, after preliminary drying in chamber. The standard moisture testing method used in gene banks is the hot-air oven method and the procedure recommended for each crop by the International Seed Testing Association is duly followed. Once the desired moisture is achieved (as mentioned below), the seeds are packed in aluminum foil packets. They have the advantage that they can be resealed and also occupy less space than other containers.

Conservation of genetic resources is carried out through two types of collections:

- Collection of seed samples for long term conservation, which is known as base collection. Base collections are maintained at –18 to –20 °C, to ensure seed viability for maximum possible time period. The moisture content of seed to be stored as base collections should be between 3 and 7 % depending on the species.
- Collection of seed samples for immediate use, termed as active collection. Active collection are maintained in conditions that ensure at least 65 % viability for 10–20 years. The moisture content of seeds to be stored as active collections should be between 3 and 8 % for seeds having poor storability and between 7 and 11 % for seeds having good storability, depending on the temperature used for storage.

These collections are conserved in different types of storage facilities. Depending on the duration of storage, three basic types of storages are recognized:

- Short term storage. The period for short term storage of seeds is from one year upto 18 months. It requires a cool and dry atmosphere (20–22 °C and 45–50 % RH) where the seeds can be conveniently stored for one to two years without much loss in their viability.
- Medium term storage. The active collection, which are generally larger than those meant for base collection are conserved in the medium term storage. The accessions are for regular distribution and therefore the time period for storage is not more than 5 yrs. The active collections are stored at temperatures ranging from 0–10 °C and relative humidity of 20–30 %.
- Long term storage. This is the storage facility for base collection, where the seeds that meet all the above mentioned criteria for NGB conservation are maintained at –18 to –20 °C.

The ex situ genebank at NBPGR comprises 12 long term modules holding the base collection. The active collections are distributed in 22 medium term modules maintained at 4 °C for storing germplasm at active sites. The genebank is currently being upgraded with new infrastructure and all efforts are being made to bring the NGB to global standards.

**Crop wild relatives**

Crop wild relatives (CWR) are wild taxa closely related to crop plants, including wild progenitors and/or wild forms of crops. Maxted et al. (2006) defined a CWR as a wild plant taxon that has an indirect use derived from its close genetic relationship to a crop. The closer the species related, the more the possibility/ practicality to get their traits incorporated. They form an important source of useful traits such as agronomic, quality, biotic and abiotic stresses, which are identified as critical component for food security and environmental sustainability in the 21st century. CWRs are often associated with disturbed habitats and neither these habitats are offered adequate protection by ecosystem conservation agencies (Maxted, Kell, 2009) nor their diversity properly conserved ex situ. CWR diversity, like that for many species, is at a declining stage; which is associated with the loss of genetic diversity (Hopkins, Maxted, 2010). This necessitates the need to establish CWR inventories which is also an indispensable tool for exploration, surveys and collection of CWR. Therefore, the need for novel genes for developing climate resilient varieties, increasing pressure on wild species populations and habitats and the present meagre ex situ collections, all accentuate the importance of locating and collecting germplasm of wild relatives.

Arora and Nayar (1984) reported the occurrence of over 320 wild relatives of crops (51 cereals and millets; 31 grain legumes; 12 oilseeds; 24 fibre plants; 27 spices and condiments; 109 of fruits, 54 of vegetables and 27 of others) in India. The NHCP of ICAR-NBPGR serves as a nodal point for confirming the botanical identity of crop wild relative’s taxa. With the identification of diversity-rich spots, availability of location details of intended taxa, India is moving forward in the systematic collecting of CWR from diverse habitats for conservation and sustainable use. Only one third of shortlisted taxa have been assembled by ICAR-NBPGR; among them more than half the taxa with <10 accessions. Analysis of gaps in collection in a scientific manner (keeping in view the conserved material, actual variability/diversity present in habitats, best utilization of GIS tools) through a mission-mode approach is currently being employed the way. In addition, detailed studies on habitat ecology, floral biology and breeding system, crossability (with crop), seed dormancy and storage behaviour of species would enable their meaningful conservation and sustainable utilization. Crossability studies aids in realization of gene-pool concept in crops, and knowing the closer relatives (even from different genera). Ensuring correct taxonomic identity, safe conservation and supply of germplasm to crop-based institutes would strengthen the pre-breeding/base-broadening/ gene-pyramiding activities through designing suitable long term multi-parental breeding programmes. All these indicate the need for trained expertise in classical subjects like taxonomy and cytogenetics, with long-term commitment. Also it is imperative to undertake studies on assessing the gene flow between wild (progenitors and naturally crossable relatives) and cultivated taxa in the wake of concerns of biosafety. All taxonomic related species may not have an equal potential as a gene donor to crops (Maxted et al., 2007). Prioritization of CWRs for management preferably on genetic relationship is important for optimization of resources. Economic importance of the crop, crossability relationship, threat and rarity of the taxa and habitat, conservation status in the genebank are the other criteria for prioritization.

Conservation of niche-specific taxa needs attention as they are often rare and endemic. Predicted extinction of species is more likely to affect RET taxa. Various steps involved in the effective management of CWRs such as development of an inventory, prioritization of CWRs taxa and habitats, ecogeographic and genetic analysis of CWRs, threat analysis and genetic erosion assessment of individual CWRs taxa, gap analysis and fixing conservation targets, development of ex situ/in situ strategies, leading to conservation and finally utilization and sustainable availability for crop improvement (Maxted et al., 2007) are all important in the Indian context also. Constituting specialized group in the country devoted to these aspects of CWRs may be a feasible option.

The above studies would facilitate a national-level mapping of CWR distribution after incorporating additional information from eco-geographic studies, which will help in the identification of CWR hotspots, which can be matched with existing protected area network in the country, thereby areas and taxa demanding conservation can be identified (Maxted et al., 2011). Strong networking among all the stakeholders working on characterization, evaluation and conservation is the need of the hour, as it is difficult for a single institute to collect, conserve and evaluate all the target species due to paucity of land, resources and expertise.

**PGR characterization and evaluation**

The utilization of PGR in crop improvement programs rests on identification of promising accessions. The collected or introduced germplasm is characterized and evaluated to assess its potential, by recording data on agronomic traits such as yield, quality, and tolerance to biotic and abiotic stresses. The germplasm is also evaluated for new traits using molecular tools to identify the genes to develop new varieties as per requirement of the farmers. Approximately 10,000 accessions are characterized/evaluated every year at ICAR-NBPGR and its regional stations. Till date, more than 2.35 lakhs accessions of different agri-horticultural crops have been characterized and evaluated and passport data is available.

Core sets have been developed to facilitate the enhanced utilization of germplasm. Genetic diversity in large collection has been determined using morphological and DNA fingerprinting markers. Mega programme on characterization and evaluation under the National Initiative for Climate Resilient Agriculture (NICRA) executed in collaboration with SAUs for 21,822 accessions of wheat and 18,775 accessions of chickpea. Generation, validation and utilization of genomic resources is one of the major objective of ICAR-NBPGR. These resources are utilized for value addition to the plant germplasm resources harboured in the genebank and for generating molecular profiles varieties of agri-horticultural crops. The advent of next generation sequencing with improved chemistries and lower input costs have resulted in high throughput data that could be mined for generating SSR and SNP markers.

**Application of genomic tools for PGR utilization and pre-breeding**

One of the major objectives of ICAR-NBPGR is to supply germplasm, collected indigenously or from exotic sources, to the breeders and other researchers in the country. These germplasm accessions have helped to develop improved varieties in various national programmes. Till date more than 5 lakh samples were supplied for utilization to various stakeholders for use in crop improvement programmes. Ever-increasing significance of conservation and utilization of PGR on one hand and advancements in computer technology for digitization and management of data on the other have catapulted PGR Informatics into limelight.

Genomics has provided various technologies including sequencing and re-sequencing platforms, availability of genome sequences as references, high-throughput genotyping platforms, SNP arrays, genome editing tools, etc. These technologies are shortly described here.

**Genome sequencing.** The inexpensive sequencing and resequencing technologies are the major driving forces behind increased number of assembled plant genomes of different crops including wild relatives. A single reference genome does not represent the total diversity within a species, hence, resequencing of cultivars, landraces and wild accessions is required to harness the total genetic variation and to identify the superior alleles for the target traits. Genome information availability has generated many next-generation sequencing-based platforms for allele mining and candidate genes identification. Next generation sequencing and whole-genome resequencing is required for discovery, validation, and assessment of diagnostic markers in different crops and it provides genome-wide markers. The draft genome sequences are now available in a number of crops through different genome sequencing consortia for rice International Rice Genome Sequencing Project (IRGSP 2005), pigeonpea (Varshney et al., 2014), chickpea (Varshney et al., 2018), wheat International Wheat Genome Sequencing Consortium (IWGSC 2018), etc.

The genome sequencing using NGS has resulted in large collections of functional markers which enhance gene assisted breeding, reducing the possibility of losing the desirable trait variation due to recombination. Sequencing and resequencing of populations developed in crossing programs or of natural population (germplasm) along with high-throughput phenotyping helps in identification and linking of variations in gene sequences to their phenotypes. Kim et al. (2016) reported the whole-genome resequencing of the 137 rice mini core collection, potentially representing 25,604 rice germplasms in the Korean genebank of the Rural Development Administration (RDA) based on the Nipponbare reference genome, and resequencing data yielded more than 15 million SNPs and 1.3 million INDELs. Further study of this rice mini core with phylogenetic and population analysis using 2,046,529 highquality SNPs successfully assigned rice accessions to the relevant rice subgroups, suggesting that the SNPs capture evolutionary signatures present in rice subpopulations. Similarly, a population structure analysis of 300 rapeseed accessions (278 representative of Chinese germplasm, plus 22 outgroup accessions of different origins and ecotypes) was carried out based on the 201,817 SNPs obtained from sequencing, divided accessions in nine subpopulations (Zhou et al., 2017). However, hierarchical clustering and principal component analysis showed intermingle of spring type accessions with semi-winter types pointing out towards frequent hybridization between spring and semi-winter ecotypes in China.

Sequence-based markers associated with rare elite alleles facilitate positional cloning and prebreeding. In case of PGR including landraces and wild relatives, screening of collection to be used for genomic analysis can be done based on passport data (collection site, specific traits, etc.) in combination with evaluation data. Sequencing based approaches provide opportunity to identify novel variations for a large number of genes through genotype-phenotype associations. Resequencing of large number of genotypes helps in determining process of origin, domestication, population structure and identifies lines with deleterious mutations in the genomes that can be eliminated to minimize the genetic load in the crop species as observed in case of maize (Bevan et al., 2017). NGS technologies together with precise phenotyping have been used for identification of marker trait associations in several crops, for example, rare wheat haplotypes effective against abiotic or biotic stresses were developed through introgression of useful and novel stress and quality traits’ alleles to lines derived from crosses of exotics with CIMMYT’s best elite germplasm under CIMMYT’s Seeds of Discovery (SeeD) initiative (Vikram et al., 2016). Singh et al. (2018) used next-generation sequencing, together with multi-environment phenotyping to study the contribution of exotic genomes to 984 three-waycrossderived (exotic/elite1//elite2) pre-breeding lines (PBLs) for accelerating grain yield gains using exotic wheat genetic resources.

**Molecular markers and genetic maps.** Recent developments in genome sequencing and or resequencing has resulted in development of large number of molecular markers in different crops. Availability of molecular markers linked to specific traits enhances pre-breeding efficiency and effectiveness through marker assisted selection (MAS). Molecular markers that are linked to the genes of a desired trait known as diagnostic markers can be indirectly used for selection of target traits (Xu, Crouch, 2008). A major earlier success for crop breeding using genomic markers was the marker-assisted introgression of the ethylene response factor, known as Submergence 1A (Sub1A) gene, for submergence tolerance into high-yielding commercial rice varieties which acts by limiting shoot elongation during the inundation period (Bailey-Serres et al., 2010). Riar et al. (2012) used polymorphic D-genomespecific SSR markers for analysing the cosegregation of the 5DS anchored markers (Xcfd18, Xcfd78, Xfd81 and Xcfd189) with the rust resistance in an F2 population, and mapped the leaf rust resistance gene (LrAC, a novel homoeoallele of an orthologue Lr57) on the short arm of wheat chromosome 5D. Vikal et al. (2014) used SSR markers for pyramiding of candidate genes for xa8, the resistance gene against Bacterial blight disease in elite rice varieties. Ellur et al. (2016) incorporated a novel Bacterial blight resistance gene Xa38 in variety PB1121 from donor parent PR114-Xa38 using a modified markerassisted backcross breeding (MABB) scheme.

Genomics has provided powerful approaches to understand interaction between many genes and complex signalling pathways in case of polygenic traits like resistance to abiotic and biotic stresses. In rice breeding, high-density genome maps are being effectively used in background selection integrated with foreground selection of bacterial blight resistance (xa13 and Xa21 genes), amylose content (waxy gene) and fertility restorer gene in order to identify superior lines with maximum recovery of Basmati rice genome along with the quality traits and minimum non-targeted genomic introgressions of the donor chromosomes (Gopalakrishnan et al., 2008). Quantitative trait loci (QTL) analysis of the genome linked to quantitative phenotypic traits, has yielded climate goverened QTL in diverse crop species (Scheben et al., 2016). Rodrigues et al. (2017) determined protein content and genetic divergence of twenty-nine soybean genotypes using 39 microsatellite markers from QTL regions of the trait grain protein content for plant breeding purposes. The pairs of genotypes with greater genetic distances and protein contents were selected to produce populations with higher means and genetic variances and greater gains with selection.

**Genome wide association studies (GWAS)** could overcome several constraints of conventional linkage mapping and provide a powerful complementary strategy for dissecting complex traits. GWAS make use of past recombinations in diverse association panels to identify genes linked to phenotypic traits at higher resolution than QTL analysis. GWAS has become a powerful tool for QTL mapping in plants because a broad range of genetic resources may be accessed for marker trait association without any limitation on marker availability. Different approaches used for GWAS include:

- SNP marker arrays or SNP chips approach. Discovery and tagging of new genes using GWAS or QTL analysis have now become much easier. The availability of high-density SNP marker arrays has opened a way for cost effective GWAS using natural populations. Wang et al. (2017) developed a high-throughput NJAU 355K SoySNP array and conducted GWAS in 367 soybean accessions (including 105 wild and 262 cultivated) across multiple environments and reported a strong linkage disequilibrium region on chromosome 20 significantly correlated with seed weight. Zhao et al. (2019) carried out meta-analysis GWAS using 775 tomato accessions (including wild accessions) and 2,316,117 SNPs from three GWAS panels and discovered 305 significant associations for the contents of sugars, acids, amino acids, and flavor related volatiles.
- Genotyping by sequencing (GBS) approach. As the cost of sequencing is continuously declining, GBS also known as next generation genotyping method, is becoming more common for discovering novel plant SNPs and used them for GWAS studies (Arruda et al., 2016). Kim et al. (2016) reported the whole-genome resequencing of 137 rice mini core collection and conducted genome wide association studies on four agriculturally important traits including ‘grain pericarp colour’, ‘amylose content’, ‘protein content’, and ‘panicle number’ and identify some novel alleles. Similarly, Arora et al. (2017) genetically characterized 177 A. tauschii accessions using GBS to study the variation for grain size using genome-wide association study.

**Genomic selection.** Genomics assisted breeding approach known as genomic selection (GS) is a better approach which simultaneously uses large genotypic data (genome wide) (exceeding phenotypic data), phenotypic data and modelling using statistical tools to predict the genomic estimated breeding values (GEBVs) for each individual (Meuwissen et al., 2001; Crossa et al., 2017). In genomic selection, a statistical model is generated using a representative population of the breeding population known as training population. This model is subsequently used to calculate the allelic effects of all marker loci, i. e. genomic assisted breeding values without having phenotypic data and these values can be used for preselection of trait-specific genotypes (Heffner et al., 2011). Xu et al. (2012) and Spindel et al. (2016) highlighted that coupling of genome wide data with genomic selection offered great specificity and predictability which can be used to accelerate prebreeding. Using GS, complex traits can be improved rapidly through generation of reliable phenotypes by shortening the selection cycle. GS application in pasture grass Lolium perenne resulted in four-year reduction in the breeding cycle (Lin et al., 2016). In genomic selections, genomic estimated and true breeding values were found to be closely correlated, even for polygenic traits with low heritability (Jia, Jannink, 2012). GS can facilitate selection of complex traits, e. g., grain yield (Saint Pierre et al., 2016) and tolerance to abiotic and biotic stress.

In genomic selection, genetic diversity specific to the population or family (species) of interest is captured through markers developed through GBS which minimized the ascertainment bias. GS is superior in respect of fixing all the genetic variation and to select individuals with higher Genomic Breeding Value (GEBV) without any phenotyping. In case of polyploid species, polysomic inheritance and possibility of double reduction requires specific consideration while using for genomic selection.

**Genome editing.** Recent advancements in genomics have also made feasible the editing of genomes and their use in crop improvement programs. Pre-breeding involves genetic transformation through recombination and genome editing (GE) tools provide an alternative. To replace conventional genetic engineering, a number of genome editing technologies have been developed during last two decades including antisense, RNA interference (RNAi), virus-induced gene silencing (VIGS), oligonucleotide directed mutagenesis (ODM), zinc finger nuclease (ZFN), transcription activator-like effects nucleases (TALENs), and clustered regularly interspaced short palindromic repeats/Cas9 (CRISPR/Cas9) (Sauer et al., 2015). These genome editing technologies can accelerate pre-breeding programs through beneficial knockout mutations, e. g. identification of genes for disease resistance or suppressing of unwanted traits linked with desired traits in wild species, as products of these technologies are not considered a genetically modified organism (GMO) (Huang et al., 2016). Most of these genome editing tools except RNAi, act by inserting, removing or replacing specific regions of genome with the help of specific nucleases known as “molecular scissors” (Esvelt, Wang, 2014).

GE approaches can be used to modify genes with defined quantitative trait nucleotides (QTNs) that cause a sizeable phenotypic effect. Further, GE tools can be used for broadening the allele pool through generating targeted variations useful for genomic selection (Scheben, Edwards, 2017; Scheben et al., 2017). Using GE tools, desired traits can be physically linked to ensure their co-segregation known as “trait stacking” (Urnov et al., 2010). For example, ZFN-assisted gene targeting helped insertion of heritably insert herbicide-resistant genes (SuRA/SuRB and PAT) in the Z. mays genome (Shukla et al., 2009). Zhang et al. (2016) recently used CRISPR/Cas9 system for production of homozygous transgene free wheat mutants. Jenko et al. (2015) showed that GE and GS, both can be combined and referred as the promotion of alleles by genome editing (PAGE) which also has a great potential for pre-breeding. Recently, Gupta (2019) reviewed the latest modification of CRISPR/Cas9 system, a base editing technology applicable to DNA as well as RNA, has revolutionized GE and demonstrated in several crops including rice, maize, wheat, etc. will be highly useful for base broadening to be used for genomic selection and relating phenotypes to genes through mutant development, particularly in primitive landraces and wild species and will provide a new direction to pre-breeding programmes.

**PGR Informatics**

PGR Informatics is the management (creation, storage, retrieval and presentation) and analyses (discovery, exploration and extraction) of diverse information (facts, figures, statistics, knowledge and news). PGR Informatics has assumed significance because of the following factors: (i) increased awareness about PGRFA, (ii) various international agreements (CBD, GPA, ITPGRFA) coming into force, (iii) availability of information in text, images, maps, videos, etc., (iv) technologies to record, link and archive such diverse types of information, (v) growing power (and falling costs) of computers and internet to facilitate access and retrieval. Fundamental merit of an organized digital information system is that it provides fair and just opportunity for all to access. On-line portals, as a consequence of PGR Informatics, enable non-exclusive access to PGR information to a large number of users involved in overlapping research areas on PGR management. Typically information is collected on details of multitude of passport data including taxonomy, biogeography, and ethnobotany of the germplasm acquisitions (domestic collections and exotic introductions), their seed health, multiplication for supply and conservation, regeneration, experimental data on characterization and evaluation leading to utilization. In addition to field data, it also includes biochemical and genomic data as well as publications. Once the information is digitized and stored, computer technologies allow management and analysis irrespective of the scale and types of data leading to better visualization and predictions.

The need for countries to develop, maintain and exchange information “from all publicly available sources, relevant to conservation and sustainable use of biological diversity” including “results of technical, scientific and socio-economic research” has been recognized in the Convention on Biological Diversity (CBD, Articles 7d, 17), and the Global Plan of Action (GPA, priority activities 17 and 18). Information of this nature is imperative for planning and implementing activities; sustainable use and sharing of benefits accrued from its use. Global assessment indicates that many of the world’s PGR are insufficiently and poorly documented. The passport information and characterization and evaluation data on genebank accessions conserved in genebanks are either lacking or poorly recorded or scattered at different places, such as passport data sheets, reports of collection and exploration missions, crop catalogues, published articles, etc. In addition, there exist informal or non-coded knowledge held by traditional farmers and indigenous people. To use this information efficiently and effectively, valuable information needs to be collected, collated, maintained and exchanged with the help of PGR Informatics.

Important PGR Informatics applications developed and maintained at NBPGR are:

- PGR Portal pgrportal.nbpgr.ernet.in
- Import Permit and EC Data Search exchange.nbpgr.ernet.inexchange.nbpgr.ernet.in
- Genebank Dashboard genebank.nbpgr.ernet.ingenebank.nbpgr.ernet.in
- PGR Map pgrinformatics.nbpgr.ernet.in/pgrmap 5
- National Herbarium of Crop Plants pgrinformatics.nbpgr.ernet.in/nhcp
- Biosystematics Portal pgrinformatics.nbpgr.ernet.in/cwr
- PGR Climate pgrinformatics.nbpgr.ernet.in/pgrclim
- PGR and IPRs http://pgrinformatics.nbpgr.ernet.in/ip-pgr/

**Recent advances in PGR Informatics in India**

NBPGR has been striving to establish PGR information set up since 2002 (Archak, Agrawal, 2012). Development of mobile apps in PGR Informatics facilitates enhanced access to PGR information which in turn could lead to enhanced utilization. NBPGR has developed two mobile apps “Genebank” and “PGR Map”. Both the apps are first of their kind for any genebank in the world. The apps have been developed for both Android and iOS. No other ICAR app is available for iPhone. Licenses were purchased and the apps have been hosted on Google Play and App Store.

Genebank app provides a dashboard view of indigenous collections (state-wise), exotic collections (country-wise), addition of accessions to genebank, etc. The app also helps generate routine genebank reports. The app uses databases live on the backend and hence always gives updated information.

PGR Map app offers three benefits: “What’s around me” helps user to obtain quickly the accessions that have been collected and conserved in the genebank from a particular location in India where the user is located at the moment; “Search the map” helps user to list the accessions that have been collected and conserved in the genebank from any selected location in India; “Search for species” helps user to map the collection sites of a crop species.

**Perspectives**

All countries are interdependent for their PGR requirements and cannot acquire and conserve resources to satisfy all their needs. There is a need to collaborate at local, regional and international levels for the acquisition and conservation of the germplasm. It is also imperative to obey the quarantine and biosafety rules for the safe movement of germplasm. Priority collection trips are required to identify areas which are not sufficiently covered so far and a repeated visits are required to be made in areas that showed diversity in the past. New tools of geographical information system (GIS) and remote sensing need to be deployed to supplement the existing ground data in PGR programmes to exploit agro-biodiversity particularly in difficult/inaccessible areas.

There is a need to adopt complementary conservation strategies involving both in situ and ex situ approaches. For in situ conservation due attention is required to be given to genetically rich hotspots including tribal belts and to strengthen and expand the network of germplasm conservation by including all the stakeholders, including the communities. Characterization and evaluation are essential to promote the utilization of materials. These tasks require substantial inputs and a decentralized evaluation network. There is a need to modify the descriptors for evaluation accordingly, and make the search for the desired characteristics in the database as quick and efficient as possible. The core collection concept is more structured and efficient approach to identify limited sets of diverse germplasm and utilise the same more effectively. Pre-breeding is needed to incorporate new kinds of pest resistance, to bring in new levels of productivity and stability of performance, and to provide quality traits for food and feed products. Awareness generation of the people at various levels (policy makers, scientific, administration, farmers, etc.) about the value of PGR wealth, its protection and conservation is essential. In addition the interface among different stakeholders is likely to bring out new useful PGR management alternatives.

## Conflict of interest

The authors declare no conflict of interest.
